# Impact of gastroesophageal reflux disease severity on dental caries and erosive tooth wear: a case control study

**DOI:** 10.1186/s12903-026-08940-0

**Published:** 2026-07-11

**Authors:** Ahmed Noaman Ali, Eman A. Tawfik, Mai Aboelmaaty, Mohamed Hamdy Sayed, Muhammad Shahzad, Mohamed A. Tawfik, Al-Zahraa M. El-Marhomy

**Affiliations:** 1 Department of Basic Dental Sciences, Faculty of Dentistry, Zarqa University, Zarqa, Jordan; 2 Oral Pathology Department, Faculty of Dentistry, Tanta University, Tanta, Egypt; 3 Dental Biomaterials Department, Faculty of Dentistry, Tanta University, Tanta, Egypt; 4 Endodontics Department, Faculty of Dentistry, Suez Canal University, Ismailia, Egypt; 5 Department of Basic Medical Sciences, Faculty of Dentistry, Zarqa University, Zarqa, Jordan; 6 Hepato-Gastroenterology and Internal Medicine Department, Faculty of Medicine, Tanta University, Tanta, Egypt; 7 Restorative Dentistry Department, Faculty of Dentistry, Tanta University, Tanta, Egypt

**Keywords:** GERD, DMFT, BEWE, PI, Salivary pH, Barrett's Esophagus

## Abstract

**Background:**

Gastroesophageal reflux disease (GERD) is linked to several extraesophageal complications, including adverse effects on oral health. This study evaluated the relationship between GERD severity, dental caries, and erosive tooth wear.

**Methods:**

A cross-sectional case–control study was conducted between January and June 2025 at Tanta University Hospital, Egypt, involving adults with GERD (*n* = 100), diagnosed by upper gastrointestinal endoscopy, and age- and gender-matched healthy controls (*n* = 100). GERD severity was classified using the Los Angeles (LA) system. Dental caries and enamel defects were assessed using the DMFT index, BEWE score, and plaque index. Salivary pH, flow rate, and routine laboratory parameters were also measured. Associations between GERD severity and oral health indicators were examined using Chi-square tests, Fisher’s exact tests, ANOVA, and multivariate regression analysis.

**Results:**

GERD patients reported significantly higher intake of acidic beverages, greater use of proton pump inhibitors, and more frequent dry-mouth symptoms. They also showed a significantly higher prevalence of dental caries and erosive tooth wear, along with lower salivary pH and flow rates compared with healthy controls (*p* < 0.001). The severity of GERD (Grades A–D and Barrett’s esophagus) was positively correlated with worsening oral findings. Logistic regression identified GERD severity and low salivary pH as independent risk factors for erosive tooth wear.

**Conclusions:**

GERD is significantly associated with increased dental caries and erosive tooth wear, particularly in individuals with more advanced disease and Barrett’s esophagus. These findings support the need for routine oral health screening and preventive care in patients diagnosed with GERD.

**Supplementary Information:**

The online version contains supplementary material available at 10.1186/s12903-026-08940-0.

## Introduction

Gastroesophageal reflux disease (GERD) is a well-known gastrointestinal disorder characterized by recurrent reflux of gastric contents into the esophagus and sometimes, into oral cavity. It is a common public health issue affecting around 1.02 billion individuals worldwide especially in developing countries [1, 2]. While the primary diagnosis of GERD has traditionally been focused on esophageal symptoms like heartburn, regurgitation, and chest discomfort, increasing attention is now being given to extra-esophageal manifestations such as chronic cough, laryngitis, ENT problems, and more recently, oral health problems [3–5]. Among these, GERD associated oral health issues has received considerable attention from researcher across the world in recent years.

Dental caries and erosive tooth wear represent two distinct oral pathologies with fundamentally different etiological mechanisms. Dental caries is a biofilm-mediated, sugar-driven disease resulting from bacterial metabolism of fermentable carbohydrates, leading to localized acid production and progressive demineralization of tooth structure. Salivary flow rate, buffering capacity, and alterations in the oral microbiota play central roles in modulating caries risk, and systemic conditions that impair these protective factors may predispose individuals to increased caries prevalence [6, 7].

In contrast, erosive tooth wear is a chemically mediated process characterized by direct dissolution of dental hard tissues by acids not derived from bacterial metabolism. Acidic challenges may be extrinsic, such as dietary acids, or intrinsic, most commonly gastric acid associated with (GERD). Repeated exposure of the dentition to gastric contents in GERD patients has been strongly associated with the development and progression of erosive tooth wear, particularly on palatal and occlusal surfaces [8–11].

GERD may therefore influence oral health through multiple biological pathways, affecting both dental caries and erosive tooth wear. Reflux-related alterations in salivary flow rate, pH, and oral microbiota may increase susceptibility to dental caries [9–13], while direct exposure of teeth to gastric acid contributes predominantly to erosive tooth wear [8–9]. Accordingly, the present study investigated both dental caries and erosive tooth wear to provide a comprehensive assessment of oral health outcomes in relation to GERD severity.

While there is considerable research evidence linking (GERD) with oral health [14, 15], its association with certain conditions such as dental caries remains inconclusive [16]. Methodological variations, differences in population characteristics, inadequate control for confounding variables, and lack of detailed clinical examinations are among the most important reasons for inconsistent findings. The majority of existing studies also lack a thorough investigation of how GERD severity affects dental outcomes, often relying on self-reported reflux symptoms rather than objective diagnostic methods such as endoscopy. Important questions, such as whether mild GERD poses the same dental risk as Barrett’s esophagus, therefore remain largely unanswered [17]. The current study aims to address this research gap by exploring the association between GERD severity and dental caries and erosive tooth wear using a case–control study design.

## Methods

### Study design and setting

This cross-sectional, analytical study was conducted at the Internal Medicine Department, and the affiliated outpatient dental clinic of the Tanta University Egypt from January and June 2025. The study followed the ethical principles outlined in the declaration of Helsinki. The institutional ethical committee at Tanta University (ID: 36264PR1280/7/25) approved the study protocol. Written informed consent was obtained from all participants prior to enrollment. A convenience sampling strategy was used, enrolling all eligible patients presenting during the study period. No formal a priori sample size calculation was performed.

### Inclusion and exclusion criteria

Inclusion criteria for the study were adult patients of any gender, aged 18 to 65 years and diagnosed with GERD through upper gastrointestinal endoscopy. Age and gender matched healthy controls with no signs and symptoms, or clinical diagnosis of GERD were also recruited. Participants with chronic oral or systemic diseases requiring the use of medication, diagnosed with xerostomia or salivary gland disorders, recent antibiotic use, a history of head and neck radiation therapy, pregnancy or lactation, or glycated hemoglobin levels over 7% were also excluded. A total of 200 participants, including 100 GERD patients and 100 healthy controls were recruited.

### Collection of demographic and oral health data

Sociodemographic and oral health data were collected using an interviewer-administered, structured questionnaire specifically developed for the current study. The questionnaire included demographic information, oral hygiene practices, dietary habits, lifestyle factors such as smoking, and medical data including current proton pump inhibitor use and duration of use categorized as < 6 months, 6–12 months, and > 1 year, duration was documented descriptively and was not included in the primary analysis. The full questionnaire is provided as supplementary material.

### GERD assessment and classification

GERD severity was assessed through upper gastrointestinal endoscopy by a single trained gastroenterologist. The Los Angeles Classification system [18] was employed to grade GERD severity from Grade A to Grade D based on mucosal breaks and erosive changes. Barrett’s esophagus cases were confirmed through histopathological examination of endoscopic biopsies when clinically indicated.

### Clinical oral examination

Comprehensive oral examinations of all participants were conducted by three calibrated dentists using WHO approved, standardized protocols. Three validated indices were employed to assess different aspects of oral health status. Caries status of the teeth was assessed using the widely used DMFT index [19]. The DMFT Index was calculated as the sum of decayed, missing, and filled teeth, with scores ranging from 0 to 28 or 32, depending on the total number of teeth present. The Basic Erosive Wear Examination (BEWE) score [20] was used to evaluate erosive tooth wear, with each of the six oral sextants scored based on the most severely affected tooth surface. Erosive tooth wear was graded from 0 (no erosive wear) to 3 (hard tissue loss of 50% or greater of the surface area), with cumulative scores categorized into risk bands ranging from no risk (0–2) to high risk (≥ 14). The Plaque Index (PI) [21] was assessed by examining four surfaces per tooth (buccal, lingual, mesial, and distal) and scoring plaque thickness from 0 (no plaque) to 3 (heavy plaque accumulation). The final scores were calculated by dividing total plaque scores by the number of surfaces examined.

### Salivary analysis

Unstimulated whole saliva samples were collected under standardized conditions to assess salivary pH and flow rate. Participants were instructed to sit upright and relax for five minutes before collection. Saliva samples were collected in the morning. Participants refrained from eating, drinking, or tooth brushing for at least one hour prior. Saliva was accumulated in the mouth without stimulation and expectorated into sterile tubes every 60 s for five minutes. Salivary pH was Salivary pH was measured immediately using a calibrated digital pH meter using a small aliquot of the collected sample. The unstimulated saliva flow rate was calculated by dividing the total volume collected by the collection time, with normal values defined as greater than 0.3 mL per minute, low values as 0.1–0.3 mL per minute, and hyposalivation as less than 0.1 mL per minute [22]. 

### Statistical analysis

Statistical analysis was performed using SPSS software version 29 (IBM Corp., Armonk, NY, USA). The normality of data distribution was assessed using the Shapiro–Wilk test and visual inspection of histograms. Quantitative parametric data were expressed as mean ± standard deviation (SD) and compared between two groups using the unpaired Student’s t-test, or among more than two groups using one-way analysis of variance (ANOVA). Qualitative variables were presented as frequencies and percentages and analyzed using the chi-square test or Fisher’s exact test, as appropriate.

To identify factors associated with severe erosive tooth wear, a multivariable logistic regression model was applied. The dependent variable was severe erosive tooth wear, defined as a Basic Erosive Wear Examination (BEWE) score greater than 9. Independent variables included age, sex, smoking status, proton pump inhibitor use, salivary flow rate, salivary pH, GERD severity based on endoscopic findings, and relevant oral hygiene and dietary factors. Variables with a p-value < 0.20 in the univariable analysis were eligible for inclusion in the multivariable model. Adjusted odds ratios (ORs) with 95% confidence intervals (CIs) were reported. A two-tailed p-value ≤ 0.05 was considered statistically significant.

## Results

### Demographic characteristics and oral hygiene habits of the participants

Table 1 present the demographic characteristics, oral health and hygiene habits of the participants. Overall, no significant differences were present between GERD and healthy controls in terms of age, gender and tobacco use (smoking). However, the BMI and consumption of acidic beverages was significantly high in GERD group compared to healthy controls. Similarly, the PPI use and self-reported dry mouth symptoms was also more frequent in GERD patients.

**Table 1:** Demographic data of the studied groups

Variable	GERD Group (*n* = 100)	Control Group (*n* = 100)	*p*-value
Age (years)	42.3 ± 12.1	41.7 ± 11.8	0.642
Gender (Male %)	56%	53%	0.711
BMI (kg/m²)	28.9 ± 3.5	26.4 ± 3.1	0.004*
Acidic beverage intake ≥ 3/week	61%	29%	< 0.001*
Smoking	29%	25%	0.549
Tooth brushing frequency < 2/day	63%	42%	0.006*
Use of fluoride toothpaste	64%	70%	0.329
Current PPI use	89%	12%	< 0.001*
Self-reported dry mouth	48%	17%	< 0.001*

Data presented as mean ± SD or (%)

*BMI* Body mass index, *PPI *Proton pump inhibitors

*Statistically significant at *p* < 0.05

### Oral health status

Clinical oral health status as assessed by DMFT, BEWE and PI score as well as lab-based salivary parameters (pH and flow rate) were further compared between GERD patients and their healthy counterparts. Using independent sample t-test, we found high DMFT, BEWE and PI in GERD patients compared to the healthy controls and the differences were statistically significant (Table 2). In contrast, salivary pH and flow rate was significantly lower in GERD patients.

**Table 2:** Oral health status of the participants

Variable	GERD Group (*n* = 100)	Control Group (*n* = 100)	*p*-value
DMFT score	8.6 ± 2.4	5.1 ± 1.8	< 0.001*
BEWE score	10.3 ± 3.1	6.2 ± 2.7	< 0.001*
Plaque index	1.9 ± 0.5	1.4 ± 0.4	0.002*
Salivary pH	5.8 ± 0.3	6.6 ± 0.2	< 0.001*
Salivary flow (ml/min)	0.34 ± 0.09	0.47 ± 0.08	< 0.001*

Data presented as mean ± SD or (%)

*DMFT* Decayed, missing and filled teeth, *BEWE* Basic erosive wear examination

*Statistically significant at *p* < 0.05

### Impact of GERD on oral health status of the participants

**Fig. 1:**
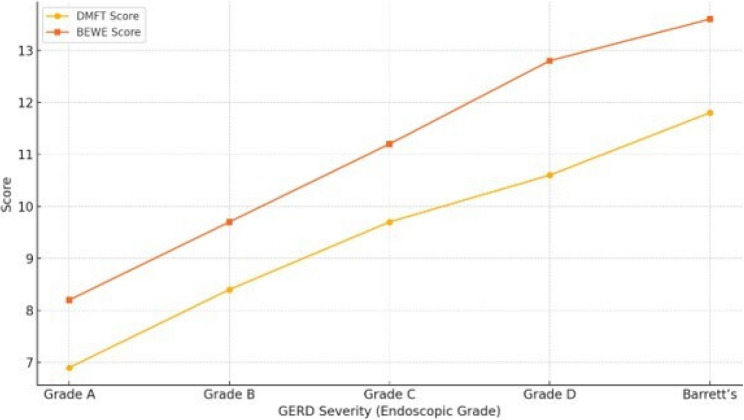
Relationship between DMFT and BEWE scores and GERD severity

We next assessed how oral health parameters changes with GERD severity. One-way ANOVA analysis revealed oral health parameters worsened with GERD severity (Table 3; Fig. 1). For example, DMFT and BEWE score gradually increased with GERD severity with Barrett’s esophagus patients had significantly higher (*p* < 0.001) DMFT and BEWE scores while the patients with grade A GERD severity had the lowest scores. In contrast, salivary pH was highest in GERD patients with Grade A severity and lowest in Barrett’s esophagus patients (*p* < 0.001).

**Table 3:** Association between oral health parameters and GERD severity of the studied groups

GERD severity (Endoscopic)	*n*	DMFT score	BEWE score	Salivary pH
Grade A	22	6.9 ± 1.7	8.2 ± 2.4	6.1 ± 0.2
Grade B	36	8.4 ± 2.1	9.7 ± 2.5	5.9 ± 0.3
Grade C	28	9.7 ± 2.6	11.2 ± 2.8	5.6 ± 0.3
Grade D	10	10.6 ± 2.9	12.8 ± 3.0	5.4 ± 0.4
Barrett’s esophagus	4	11.8 ± 1.5	13.6 ± 2.2	5.2 ± 0.2
p-value (ANOVA)		< 0.001*	< 0.001*	< 0.001*

Data presented as mean ± SD

*GERD* Gastroesophageal reflux disease, *DMFT* Decayed, missing and filled teeth, *BEWE* Basic erosive wear examination

*Statistically significant at *p* < 0.05

### Predictors of enamel erosion in GERD patients

Multivariable logistic regression analysis identified factors independently associated with severe erosive tooth wear. The analysis showed that GERD was strongly associated with enamel erosion (BEWE > 9), with higher odds observed in affected patients, while severe GERD (Grade C/D) and low salivary pH were also significantly associated with increased likelihood of erosive tooth wear (Table 4). Acidic beverage intake was associated with a higher probability of erosion, whereas brushing frequency showed no significant association. Overall, multiple factors, including reflux-related acid exposure, salivary acidity, and dietary habits, appear to contribute to the observed patterns of erosive tooth wear.

**Table 4:** Multivariable logistic regression analysis of factors associated with severe erosive tooth wear (BEWE > 9)

Predictors	Odds ratio (OR)	95% confidence interval	*p*-value
GERD diagnosis	4.75	2.33–9.67	< 0.001*
GERD severity (Grade C/D)	3.58	1.72–7.45	0.001*
Salivary pH < 6.0	3.21	1.56–6.61	0.001*
Acidic beverage intake	2.44	1.19–5.02	0.015*
Poor brushing (< 2/day)	1.73	0.89–3.37	0.104

*GERD* Gastroesophageal reflux disease

*statistically significant at *p* < 0.05

## Discussion

The present study demonstrated a significant association between GERD severity and oral health outcomes, including dental caries and erosive tooth wear. An increasing trend in DMFT and BEWE scores was observed across GERD severity grades, extending from Los Angeles Grade A esophagitis to Barrett’s esophagus; however, findings related to Barrett’s esophagus should be interpreted cautiously due to the limited sample size of this subgroup. Overall, these results suggest an association between gastric acid exposure and alterations in dental hard tissues, although this relationship must be interpreted in light of differences in dietary habits, oral hygiene practices, and medication use between the study groups. Our findings are consistent with previous reports describing oral manifestations associated with gastroesophageal reflux disease [21, 23].

Salivary analysis revealed significant reductions in both flow rate and pH among GERD patients. These alterations were consistently observed across disease severity categories, supporting a potential contributory role of compromised salivary function in oral health deterioration. Similar observations have been reported by Muñoz et al. [24] and Ganss et al. [25], underscoring the critical protective role of saliva in maintaining oral homeostasis. Reduced buffering capacity and salivary volume may impair natural defense mechanisms against acid challenges, particularly in the presence of frequent reflux episodes. Comparable associations have also been described in pediatric populations by da Silva AP et al. [26], suggesting that the interaction between reflux and salivary dysfunction is not limited to adulthood. Nevertheless, the role of salivary alterations should be interpreted cautiously, given the multifactorial nature of oral diseases and the cross-sectional design of the present study.

Furthermore, the observed association between reflux severity and dental damage aligns with reports of increased dental sensitivity and reduced salivary pH among GERD patients [27, 28], collectively supporting the broader concept of reflux-associated oral health impairment.

Beyond direct acid exposure, pepsin has been increasingly recognized as a potential mediator of reflux-related oral tissue damage. Pepsin is a proteolytic enzyme that may remain active at weakly acidic and neutral pH levels and has been shown to degrade the acquired salivary pellicle, thereby facilitating direct acid contact with enamel surfaces. Disruption of this protective pellicle may compromise the efficacy of bioactive salivary components and increase enamel susceptibility to erosive challenges. Recent experimental and clinical evidence supports this mechanism, highlighting pepsin as an additional contributory factor in reflux-associated erosive tooth wear [29]. In this context, patients with Barrett’s esophagus exhibited more pronounced oral health deterioration; however, given the small number of patients in this subgroup, these observations should be considered exploratory and interpreted with caution Beyond direct acid exposure. Prolonged exposure to gastric acid and pepsin may exert cumulative effects on dental tissues, potentially accounting for the greater severity of oral findings noted in advanced disease stages [30].

Dietary behavior also emerged as a relevant contributor to oral health outcomes in the present study. Many GERD patients reported frequent consumption of acidic beverages several times per week, a factor previously associated with erosive tooth wear [31]. Reduced tooth brushing frequency was also reported among GERD patients, although this variable did not retain statistical significance in the multivariable regression analysis. These behaviors nevertheless represent potential confounding factors that may have influenced both caries and erosive tooth wear.

Multivariable regression analysis identified GERD severity as an independent predictor of erosive tooth wear. In contrast, the association between GERD and dental caries should be interpreted cautiously, as caries outcomes were assessed using bivariate analyses and were not adjusted for key confounders such as dietary habits and oral hygiene practices. Reduced salivary pH was independently associated with erosive tooth wear, suggesting that GERD may affect oral health through complementary mechanisms, including direct acid exposure and indirect impairment of salivary protective functions. This interpretation is consistent with previously proposed mechanistic frameworks describing reflux-related oral tissue damage [32, 33].

The present study is, to our knowledge, among the first to evaluate oral health outcomes in relation to GERD severity using endoscopic classification rather than symptom-based assessment alone. The inclusion of Barrett’s esophagus as a distinct analytical subgroup further enabled exploration of advanced disease stages. Nevertheless, several limitations should be acknowledged. This was a single-center study conducted at a university hospital, which may limit generalizability. A convenience sampling strategy was used, and no a priori sample size calculation was performed. The use of the DMFT index instead of a more sensitive system such as ICDAS may have underestimated early carious lesions, and inter-examiner reliability testing was not conducted. Control participants did not undergo endoscopic or pH evaluation, and the possibility of silent GERD cannot be excluded. A small proportion of control participants reported PPI use, which may reflect use for non-GERD indications or the presence of undiagnosed or silent reflux. Salivary pepsin was not measured, limiting biochemical correlation with dental outcomes, and proton pump inhibitor use—although potentially influential—was not included in the regression model due to its high prevalence and limited variability among GERD patients. Finally, the cross-sectional design, single-time saliva sampling, and absence of 24-hour pH monitoring preclude causal inference. Despite these constraints, the overall consistency of findings across analytical approaches supports the robustness of the observed associations.

## Conclusions

Gastroesophageal reflux disease severity was significantly associated with erosive tooth wear, particularly in patients with more advanced disease stages, underscoring the impact of reflux on dental hard tissues. Although increased caries experience was observed among GERD patients, this association should be interpreted cautiously due to the influence of dietary and oral hygiene factors and the absence of multivariable adjustment for caries outcomes. These findings highlight the multifactorial nature of GERD-related oral health changes and support the need for integrated dental assessment and preventive care in affected patients.

## Recommendations

Routine dental examinations should be considered an integral component of comprehensive care for patients with GERD. Patient education should focus on maintaining adequate oral hygiene and limiting the intake of acidic foods and beverages to reduce the risk of dental complications. Future longitudinal research is warranted to better elucidate the role of salivary alterations and reflux constituents, such as pepsin, in the progression of GERD-related oral health changes.

## Supplementary Information

Supplementary Material 1.

## Data Availability

The data supporting the findings of this study are available from the corresponding author upon reasonable request.

## Abbreviations

ANOVA: Analysis of variance
BEWE: Basic Erosive Wear Examination
CI: Confidence Intervals
DMFT: Decayed, Missing, Filled Teeth
GERD: Gastrointestinal Reflux Disease
OR: Odds Ratios
PPI: Proton Pump Inhibitors
