# Supplementary material for: Impact of gastroesophageal reflux disease severity on dental caries and erosive tooth wear: a case control study
Source: BMC Oral Health. 2026 Jul 11;26:1329. doi: 10.1186/s12903-026-08940-0 (PMC13397662; doi:10.1186/s12903-026-08940-0)
Supplement: Supplementary file 1 — Supplementary Material 1. [file 12903_2026_8940_MOESM1_ESM.docx]

**Supplementary File 1: Structured Questionnaire**

Study Title: Oral Health Outcomes in Patients with Gastroesophageal Reflux Disease (GERD) 
Administration: Interviewer-administered structured questionnaire 
Target Population: Adult participants 
 **Section A: Sociodemographic Characteristics**

1. Age (years): _______

2. Gender: ☐ Male ☐ Female

3. Marital Status: ☐ Single ☐ Married ☐ Divorced / Widowed

4. Educational Level: ☐ No formal ☐ Primary ☐ Secondary ☐ University or higher

5. Employment Status: ☐ Employed ☐ Unemployed ☐ Retired

6. Socioeconomic Status: ☐ Low ☐ Middle ☐ High

**Section B: Oral Health Status (Self-Reported)**

7. Current oral symptoms (multiple allowed):

☐ Tooth sensitivity ☐ Tooth pain ☐ Bleeding gums ☐ Bad breath ☐ Dry mouth ☐ None

 8. Noticed visible tooth wear or erosion? ☐ Yes ☐ No ☐ Not sure

9. History of frequent dental caries? ☐ Yes ☐ No

**Section C: Oral Hygiene Practices**

10. Tooth brushing frequency: ☐ < once daily ☐ Once daily ☐ Twice daily or more

11. Use of fluoride toothpaste? ☐ Yes ☐ No ☐ Not sure

12. Additional oral hygiene aids: ☐ Floss ☐ Mouthwash ☐ Interdental brushes ☐ None

13. Dental visits: ☐ Only when pain ☐ Every 1–2 years ☐ Regular annual

**Section D: Dietary Habits Relevant to Dental Health**

14. Acidic food consumption: ☐ Rarely ☐ Occasionally ☐ Frequently

15. Acidic beverage consumption: ☐ Rarely ☐ Occasionally ☐ Frequently

16. Carbonated drink consumption? ☐ Yes ☐ No

**Section E: Lifestyle Factors**

17. Smoking status: ☐ Current ☐ Former ☐ Never

18. If smoker, cigarettes/day: _______

19. Alcohol consumption: ☐ Yes ☐ No

**Section F: Medical History Related to GERD**

20. Diagnosed with GERD? ☐ Yes ☐ No

21. Current PPI use? ☐ Yes ☐ No

22. Duration of PPI use: ☐ <6 months ☐ 6–12 months ☐ >1 year

23. Other chronic diseases? ☐ Yes ☐ No  If yes, specify: __________
